# Intrasalivary Thymic Carcinoma: A Case Report and Literature Review

**DOI:** 10.1007/s12105-021-01394-6

**Published:** 2021-11-22

**Authors:** Michał Kunc, Alexandra Kamieniecki, Grzegorz Walczak, Tomasz Nowicki, Bartosz Wasąg, Bogusław Mikaszewski, Dominik Stodulski, Wojciech Biernat

**Affiliations:** 1 Department of Pathomorphology, Medical University of Gdansk, Smoluchowskiego 17, Gdańsk, Poland; 2 Student Scientific Circle of Pathomorphology, Medical University of Gdansk, Gdańsk, Poland; 3 2nd Department of Radiology, Faculty of Health Sciences, Medical University of Gdansk, Gdańsk, Poland; 4 Department of Biology and Medical Genetics, Medical University of Gdansk, Gdańsk, Poland; 5 Department of Otolaryngology, Medical University of Gdansk, Gdańsk, Poland

**Keywords:** Thymic carcinoma, Squamous cell carcinoma, CD5, CD117, Immunohistochemistry, Parotid gland, Salivary gland, CASTLE

## Abstract

Ectopic thymic carcinomas are rarely diagnosed in the thyroid gland, let alone in extrathyroid tissues. In the currently available literature, only five cases of extrathyroidal malignancies with thymic differentiation have been reported as arising in the major salivary glands. A 69-year-old female presented with a slow-growing palpable mass in the left parotid gland. Fine needle aspiration biopsy suggested metastatic cancer, whereas core needle biopsy revealed high-grade squamous cell carcinoma. The patient underwent left radical parotidectomy with selective ipsilateral lymph node dissection and subsequent radiation therapy. The surgical specimen was taken for histopathological examination. Microscopically, the tumor resembled thymic carcinoma. It was composed of large nests of squamoid cells with smooth contours, focally with a syncytial growth pattern, and accompanied by abundant lymphocytes with reactive lymphoid follicles. This appearance resembled a micronodular thymic carcinoma with lymphoid hyperplasia. Moreover, the tumor displayed expression of squamous markers (p40 and p63) and markers of thymic carcinoma (CD5 and CD117). Therefore, the final diagnosis of intrasalivary thymic carcinoma was rendered. The molecular analysis including next-generation sequencing demonstrated no variants of the strong, potential, or unknown clinical significance. The patient remains disease-free at 1-year follow-up. In the current case, we comprehensively present a clinical, microscopic, molecular, and radiological picture of CD5-positive squamous cell carcinoma of the parotid. We postulate that similar cases should be designated as intrasalivary thymic carcinoma analogically to similar thyroid tumors. Our case and the limited literature data indicate they should be distinguished from conventional squamous cell carcinoma of major salivary glands due to their rather favorable prognosis.

## Introduction

Ectopic tumors demonstrating thymic epithelial differentiation are rare lesions and when they do occur they mostly develop in the thyroid gland. The current WHO classification of thyroid tumors contains three entities: ectopic thymoma, spindle epithelial tumor with thymus-like differentiation (SETTLE), and intrathyroid thymic carcinoma (ITC) [1]. The latter, also known as carcinoma showing thymus-like elements (CASTLE), is a rare tumor probably arising from ectopic thymic tissue or embryological branchial pouch remnants [2]. The 3rd branchial pouch detaches from the pharynx and separates into the thymus and parathyroids. While both migrate downwards, the thymus migrates further where the two lobes meet in the midline above the heart [3]. In the thyroid, solid cell nests show a similar immunophenotype to thymic cells and may represent precursor lesions for the neoplasms with thymic differentiation [2]. Aberrant thymic tissue is occasionally seen in other areas of the head and neck region, predominantly in children [4]. The first three cases of thymus-like malignancies in the thyroid were initially described as “intrathyroid epithelial thymoma” in 1985 by Miyauchi et al. [5]. Later, in 1990, Chan and Rosai proposed an acronym CASTLE, to designate its resemblance to thymic carcinoma [6]. Other reported names include carcinoma showing thymus-like differentiation or features, and CD5-positive thyroid carcinoma. Intrathyroid thymic carcinoma is a rare tumor, but the available data suggests it has an overall indolent clinical course [7]. Extrathyroid malignancies with thymic differentiation are extremely rare and there have only been five cases of extrathyroid ectopic thymic carcinoma described as arising in the major salivary glands. In the current report, we describe a case of CD5-positive squamous cell carcinoma with thymus-like features, and we postulate such cases should have a designated entity, such as intrasalivary thymic carcinoma analogically similar to thyroid tumors.

## Case Report

**Fig. 1:**
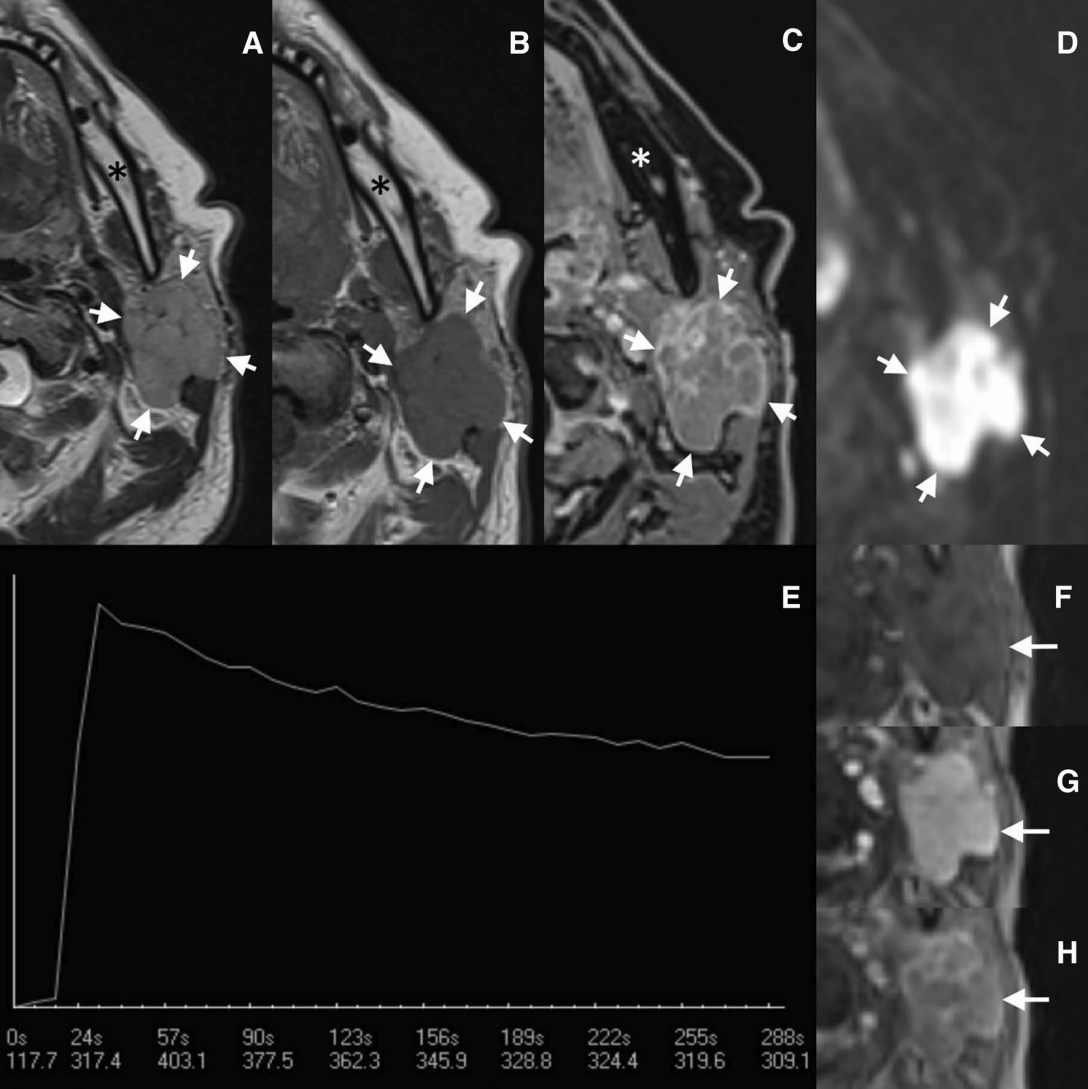
Magnetic resonance imaging in the axial plane of the left parotid region. In the turbo spin-echo sequence in T2-weighted image (**a**), the tumor (marked by the arrows) has intermediate signal intensity, observed in hypercellular lesions. In the turbo spin-echo sequence in T1-weighted image (**b**), the signal intensity is low. In the delayed contrast-enhanced gradient-echo T1-weighted image (**c**), the lesion shows heterogeneous and circumferential enhancement. In diffusion-weighted image (**d**) a marked diffusion restriction is observed (0.60–0.67 × 10^−3^ mm^2^/s), also typical for hypercellular lesions. The enhancement curve (**e**) shows the lesion’s signal intensity change after contrast agent administration on a timeline (expressed in seconds); early enhancement is visible with wash-out of the contrast agent (enhancement curve type B with wash-out ratio of 38%, typically seen in Warthin tumor). On pictures **f** to **h**, the subsequent phases of the dynamic examination are presented: pre-contrast image (**f**), early, strong, almost homogeneous enhancement of the lesion (**g**), and the last phase of the dynamic examination (**h**). Asterisk marks the mandible

**Fig. 2:**
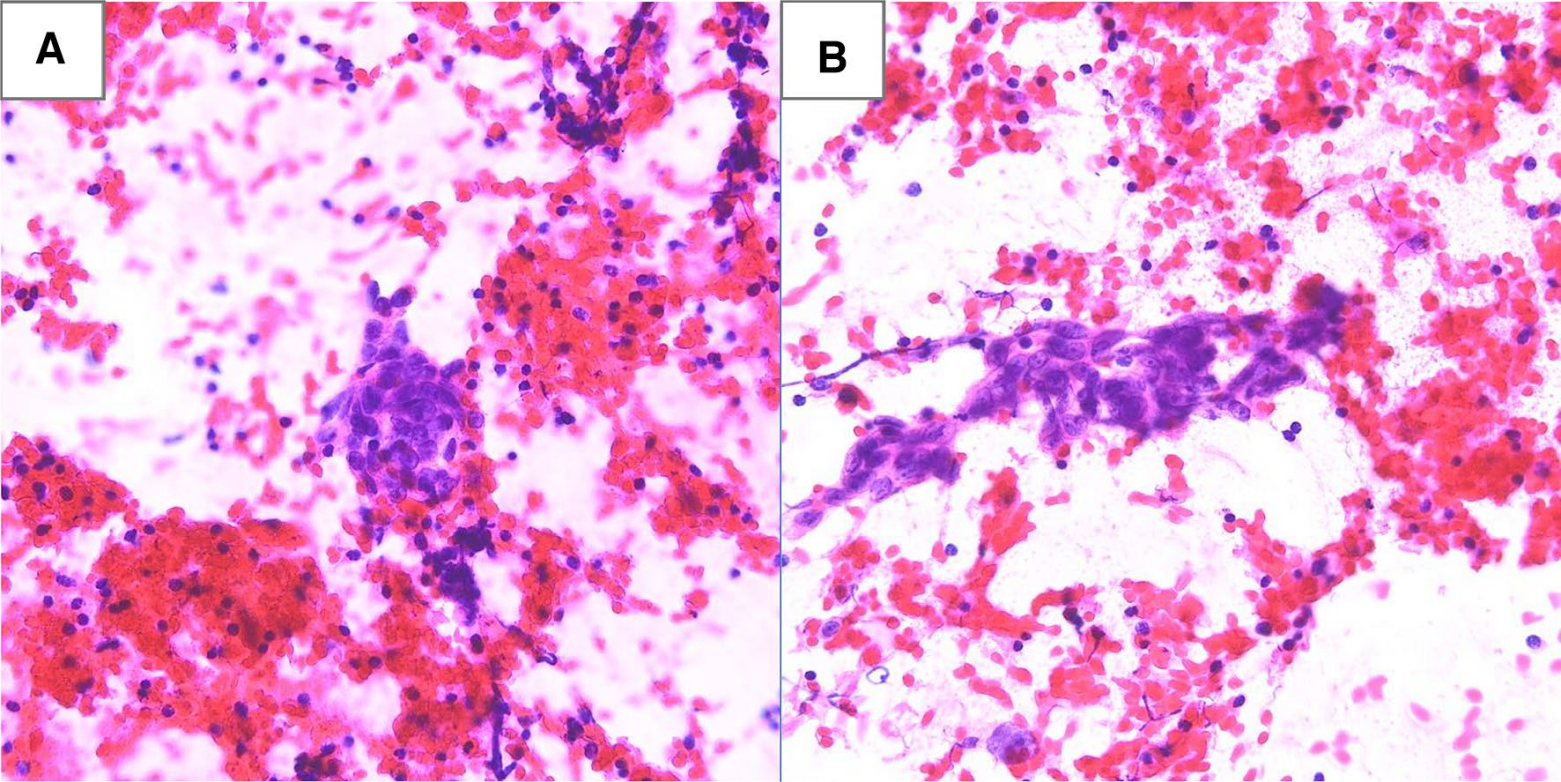
Haematoxylin and eosin stained fine needle aspiration smears from the parotid tumor. Groups of pleomorphic, epithelioid cancer cells (**A**, **B**) in the lymphocytic background

A 69-year-old Caucasian female presented with a slow-growing palpable mass in the left parotid gland that was present for the past few months. She had no other symptoms or comorbidities. A craniofacial magnetic resonance imaging revealed that in the superficial lobe of the left parotid gland, there was a well-defined, lobulated lesion bulging into the deep lobe that measured 27 × 37 × 38 mm. The lesion’s signal intensity in T2-weighted images was intermediate and in T1-weighted images, the intensity was homogenous and low. Early enhancement after intravenous contrast agent administration was seen, with features of contrast wash-out (wash-out ratio of 38%, enhancement curve type B) and a marked diffusion restriction (apparent diffusion coefficient of 0.60–0.67 × 10^−3^ mm^2^/s)—Fig. 1. Multiple enlarged, heterogenous lymph nodes in the II and VA groups on the left side were observed, with no contralateral lymphadenopathy. In the differential diagnosis of the salivary gland lesion, a lymphoproliferative process was considered (e.g., MALT lymphoma). Fine needle aspiration biopsy revealed the presence of sheets and small groups of pleomorphic epithelioid cells in the background of reactive lymphocytes (group VI according to the Milan system for reporting salivary gland cytopathology; Fig. 2). Subsequently a core needle biopsy was performed eventually leading to a diagnosis of an HPV-negative high-grade squamous cell carcinoma. Computed tomography of the chest and abdomen did not reveal any abnormalities. The patient underwent left radical parotidectomy with selective ipsilateral lymph node neck dissection (groups I–III, V). The facial nerve was partially infiltrated by the tumor and was resected during the procedure leading to facial palsy. Subsequently, the patient underwent radical radiation therapy and is currently free of the disease after 12 months of follow-up.

### Histopathological Findings

**Fig. 3:**
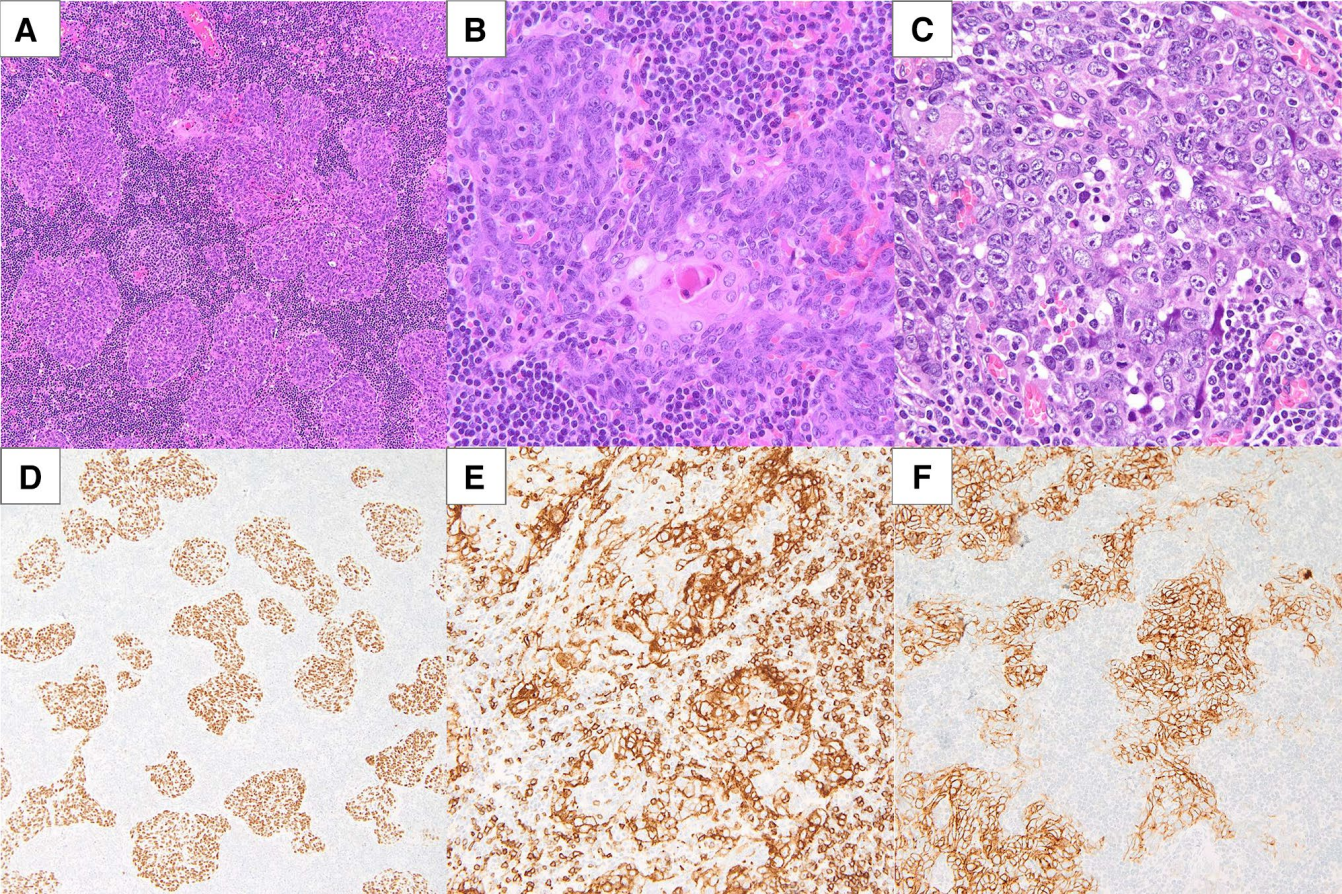
Microscopic appearance of the tumor resembling micronodular thymic squamous cell carcinoma with lymphoid hyperplasia–cohesive growth, nests of cancer cells with smooth contours, prominent nucleoli, areas of medullary differentiation, and abundant lymphoid infiltrates with reactive lymphoid follicles formation (**A**). On higher magnification a structure reminiscent of Hassall’s corpuscle is visible (**B**). In some areas cancer cells demonstrate prominent nuclear pleomorphism, conspicuous nucleoli and multiple mitotic figures (**C**). Positive p63 staining in cancer cells emphasizes the micronodular architecture (**D**). CD5 is expressed by both cancer cells and some reactive lymphocytes (**E**), whereas CD117 is expressed exclusively by squamous cell carcinoma (**F**)

Microscopic examination revealed non-keratinizing squamous cell carcinoma infiltrated by abundant lymphocytes. Tumor cells were cohesive and formed large nests with smooth contours, focally with a syncytial growth pattern and accompanied by abundant lymphocytes with reactive lymphoid follicles (Fig. 3A–C). Morphologically, the tumor resembled micronodular thymic carcinoma with lymphoid hyperplasia, a provisional subtype of thymic squamous cell carcinoma and, to a lesser extent, lymphoepithelioma-like carcinoma [8, 9]. Cancer cells showed positivity for squamous cell markers, p40 and p63 (Fig. 3D), and were negative for p16, NUT, neuroendocrine markers (synaptophysin, chromogranin, CD56), DOG-1, androgen receptor, and SOX-10. No mucin production was observed. Epstein-Barr encoding region (EBER) in situ hybridization was negative. The Ki-67 proliferative index was 30%. Due to the morphological similarities with thymic carcinoma, CD5 and CD117 stainings were performed (Fig. 3E, F). Both markers were expressed by cancer cells supporting the final diagnosis of intrasalivary thymic carcinoma (ectopic thymic carcinoma of the salivary gland). Metastasis was found in one of 69 removed lymph nodes and displayed similar morphology and immunophenotype to the primary tumor. The final pathological stage of the tumor was pT2N1 according to the 8th edition of the American Joint Committee of Cancer Staging Manual.

### Molecular Analysis

Nucleic acids were extracted from formalin-fixed paraffin-embedded tumor tissue using Cobas DNA Sample Preparation Kit (Roche Diagnostics) and FormaPure XL RNA (Beckman Coulter). Illumina TruSight Oncology 500 (TSO500) hybrid-capture assay targeting 523 cancer-relevant genes was used for comprehensive genome profiling. Library sequencing was accomplished using a NextSeq550 instrument (Illumina, San Diego, CA), and the data were analyzed using the Clinical Genomics Workspace (PierianDx). No variants of the strong, potential, or unknown clinical significance were detected.

## Discussion

Most salivary gland tumors with squamoid morphology represent mucoepidermoid carcinomas or metastatic squamous cell carcinomas of the head and neck. Primary squamous cell carcinoma arising primarily in the salivary gland is very infrequent and has unfavorable outcomes, especially in elderly patients with nodal and/or distant metastases [10]. To our knowledge, thymic-like squamous cell carcinomas in the salivary glands were reported only five times in the currently available literature. We present the 6th case of this entity arising in the major salivary glands.

Intrathyroid thymic carcinoma is one of four intrathyroid tumors with thymic differentiation and is recognized in the WHO classification of tumors of endocrine organs along with ectopic thymoma, ectopic hamartomatous thymoma, and spindle-cell tumor with thymus-like differentiation [1]. Other than ectopic cervical thymoma, the thymic nature is not immediately apparent due to the lack of immunohistochemical, ultrastructural, or genotypic thymus-specific markers [6]. However, CD5 can be a valuable marker for diagnosing thymic-like neoplasms in the thyroid, although lack of expression does not entirely rule them out [11].

The very first case of extrathyroid carcinoma with thymic-like features arising in the major salivary gland was reported in the parotid gland in a 55-year-old woman in 2017 [12]. Only one case was reported outside the parotid, in the sublingual gland of a 35-year-old man that presented with an ipsilateral neck mass and was first misdiagnosed as metastatic squamous cell carcinoma [13]. The summary of the previously published cases is presented in Table 1.

**Table 1:** Summary of the cases of ectopic thymic carcinomas arising in major salivary glands

References	Age [years]	Gender	Location	Histology	IHC	Radiology	Stage	Follow-up
[13]	35	Male	Sublingual gland	Solid cell nests separated by fibrous septa containing lymphoplasmacytic infiltrate	CK AE1/AE3 ( +) p63 ( +) CD5 ( +) CD117 (+ /−) BCL2 ( +) S100 (−) PAX8 (−) TTF-1 (−) Thyroglobulin (−) SOX-10 (−) Chromogranin (−) Synaptophysin (−)	Enlargement and enhancement of the posterior third of the right sublingual gland	pT1N1M0	24 months (NED)
[12]	55	Female	Parotid gland	Solid cell nests separated by fibrous septa containing lymphoplasmacytic infiltrate	p40( +) p63 ( +) CD5 ( +) CD117 ( +)	Well-marginated solid mass, with moderate contrast-medium affinity, in the superficial parotid, extending to the deep parotid lobe and sternocleidomastoid muscle	pT1N1M0	12 months (NED)
[15]	43	Male	Parotid gland	Solid cell nests separated by fibrous septa	CK AE1/AE3 ( +) p63 ( +) CD5 ( +) CD117 ( +)	Round soft tissue mass in the parotid gland, showing uneven and mild to moderate enhancement	N/A	10 months (NED)
[21]	23	Female	Parotid gland	Solid nests and trabeculae separated by fibrous septa accompanied by lymphoplasmacytic infiltrate	CK AE1/AE3 ( +) p40( +) p63 ( +) p16 ( +) CD5 ( +) CK5/6 ( +) LMP-1 (−)	Tumor composed of a solid portion and a cystic portion showing a slight enhancement of the solid portion and an irregular enhancement of the cyst wall Dynamic-enhanced MRI showed a short peak time and a low washout ratio	pT3N2M0	13 months (NED)
[16]	69^a^	Female	Parotid gland	N/A	CK 5/6 ( +) CD5 ( +) CD117 ( +) p63 ( +) p40 ( +)	N/A	pT1N2M0	120 months (recurred as pleural carcinomatosis treated with pembrolizumab)
Current case	69	Female	Parotid gland	Solid cell nests accompanied by abundant lymphoplasmacytic infiltrate with lymphoid follicles	CK AE1/AE ( +) CD5 ( +) CD117 ( +) p63 ( +) p40 ( +) p16 (−) Synaptophysin (− ) Chromogranin (−) CD56 (−) DOG-1 (−) AR (−) SOX-10 (−) NUT (−) EBER (−)	Lesion mainly in the superficial parotid, bulging into the deep parotid and showing early enhancement	pT2N1M0	12 months (NED)

*y* years, *IHC* immunohistochemistry, *N/A* not available, *NED* no evidence of disease

^a^Age at the time of primary tumor resection

In intrathyroid thymic carcinoma, radical resection is the treatment of choice and the prognosis after surgery is rather favorable if this treatment is achieved [14]. Just like our patient, the other intrasalivary thymic carcinomas were treated with total parotidectomy with selective neck lymph node dissection and postoperative adjuvant radiotherapy [12, 13, 15].

While intrathyroid thymic carcinoma has a generally good prognosis and it is essential to differentiate it from other more aggressive tumors [5], they may also recur after long intervals [6]. Accordingly, all patients with a salivary counterpart were alive at the date of publication, but one patient had dissemination to the pleura which occurred 10 years after removal of the primary tumor [16]. In the current report, the observation time is short (12 months) and the patient is being periodically monitored. Nevertheless, the current very limited data suggest that ectopic thymic carcinomas developing in the salivary glands demonstrate biological behavior similar to their thyroid counterparts. Due to this reason we postulate to use the analogous nomenclature for these tumors (i.e. intrasalivary thymic carcinoma).

Pathologists are aware of the existence of thymic-like neoplasms in the thyroid; thus neoplasms with thymic features are frequently considered in the differential diagnosis of thyroid malignancies. On the other hand, intrasalivary thymic carcinomas are most likely underrecognized. In one case the diagnosis was established retrospectively 10 years after the primary diagnosis [16]. We postulate that CD5 and CD117 should be included in the immunohistochemical panel for squamous cell carcinomas and undifferentiated carcinomas of the major salivary glands with an unusual microscopic features, especially if they resemble thymic carcinomas morphologically. Both markers are reliable indicators of thymic differentiation in squamous cell carcinoma. Two large studies showed that their expression in pulmonary squamous cell carcinoma is extremely rare [17, 18], and co-expression of CD5 and CD117 was not observed at all [19]. Accordingly, Ito et al. reported 100% sensitivity of CD5 expression as a marker of intrathyroid thymic carcinoma/CASTLE [5]. There is no data regarding the frequency of CD5 and CD117 expression in salivary carcinomas and this issue should be investigated in future studies.

Moreover, due to the lymphocyte-rich stroma, intrasalivary thymic carcinoma may be confused with lymphoepithelial carcinoma, which occasionally develops in the salivary glands [20]. However, the latter is usually diffusely positive for EBER in situ hybridization and is CD5 and CD117 negative. Other neoplasms which should be considered in the differential diagnosis include various carcinomas with squamous differentiation including metastatic HPV-positive squamous cell carcinoma, high-grade mucoepidermoid carcinoma, and NUT carcinoma. In the current case, the tumor was negative for p16, mucicarmine, and NUT excluding these entities from the diagnostic consideration. Nevertheless, one case of thymic carcinoma of the parotid was diffusely positive for p16 and negative for HPV DNA, which may represent a potential diagnostic pitfall [21].

The origin of intrasalivary thymic carcinoma remains elusive. It is unclear if these tumors represent thymic differentiation of salivary elements or development from ectopic thymic elements. Since the thymus is derived directly from the third pharyngeal pouch and migrates to the thorax during development, thymic tissue, and therefore also thymic tumors, can be found anywhere near its primary formation site and along its embryologic migration path [12]. Ectopic thymic elements were identified in various areas of the head and neck [22]. The molecular origin is also unclear. Comprehensive sequencing of 523 genes performed in the current case did not detect any significant variants. Similarly, sequencing of 143 cancer-related genes performed in the case described by Wong et al. revealed only germline variants of *PPARG*, *BRCA2*, and *NOTCH1* [12]. Very recently, Ishikawa et al. performed whole-exome sequencing on their case and detected five nonsynonymous and splicing somatic mutations (*FREM2* p.Val861Phe, *CLK3* p.Phe376Leu, *DLGAP1* p.Lys294Asn, *NOX1* p.Val165Met, and *PSG9* c.430+4A>T) [21]. If these molecular abnormalities are recurrent in intrasalivary thymic carcinomas remains to be elucidated in further studies.

## Conclusion

Ectopic thymic carcinoma remains a rare diagnosis, but it may involve major salivary glands. CD5 and CD117 immunohistochemistry should be considered in squamous cell carcinomas and undifferentiated carcinomas involving the salivary glands, especially when a distant primary tumor is not clinically evident and they display morphologic resemblance to thymic carcinoma. Intrasalivary thymic carcinoma should be regarded as a separate entity and should be included in the differential diagnosis of major salivary gland tumors.

## Data Availability

Not applicable.
